# Nocturnal Incontinence of Urine and Phimosis

**Published:** 1893-03

**Authors:** 


NOCTURNAL INCONTINENCE OF URINE AND PHIMOSIS.—In an article on the above subject by Dr. E. Loumeau, in the January number of *Annales de la Policlinique de Bordeaux*, the writer divides the different varieties of incontinuence under five heads:

1. Incontinence of urine of purely psychopathic origin. (Theory of T. L. Petit.)
2. Incontinence of urine due to vesical irritability. (Theory of Trousseau.)
3. Incontinence of urine due to a faulty contractility of the sphincter vesicæ, or to anesthesia of the urethra. (Theory of Guyon.)
4. Incontinence of urine due to paralysis of the bladder and sphincter.
5. Incontinence of urine due to epilesy.

Under the second head—vesical irritability—the author mentions the following causes: (1) Peripheral irritation, such as phimosis, atresia of the meatus, hypospadias, oxyurides hemorrhoids, fissures, etc. (2) Exaggerated reflex excitabllity of the cord. The author regards phimosis as the most prolific agent in this classification. Trousseau appears to have been the first to call attention to this fact in 1860, and since then Forni, Tuffier, Beard, Bouisson, Duplay, Schwartz, Birger, and many others, have called especial attention to this form of enuresis. —*Buffalo Medical and Surgical Journal.*

